# The Influence of Green Entrepreneurship on Sustainable Development in Saudi Arabia: The Role of Formal Institutions

**DOI:** 10.3390/ijerph18105433

**Published:** 2021-05-19

**Authors:** Wafa Alwakid, Sebastian Aparicio, David Urbano

**Affiliations:** 1 Department of Business, Universitat Autònoma de Barcelona, Edifici B Campus UAB, Bellaterra (Cerdanyola del Vallès), 08193 Barcelona, Spain; wafanaif.alwakid@e-campus.uab.cat; 2 Department of Business Administration, Jouf University, Al Jouf 75471, Saudi Arabia; 3 Durham University Business School, Durham University, Mill Hill Lane, Durham DH1 3LB, UK; 4 Fundación ECSIM, Medellin 050010, Colombia; 5 Department of Business and Centre for Entrepreneurship and Social Innovation Research (CREIS), Universitat Autònoma de Barcelona, Edifici B Campus UAB, Bellaterra (Cerdanyola del Vallès), 08193 Barcelona, Spain; david.urbano@uab.cat

**Keywords:** green entrepreneurship, sustainable development, entrepreneurship policy, formal institutions, Saudi Arabia

## Abstract

This study explores the influence of green entrepreneurial activity on sustainable development, using institutional economics as a theoretical framework. Also, the role of entrepreneurship policy is analysed in the context of Saudi Arabia. Using information from the General Authority for Statistics from 13 Saudi Arabian cities, the main findings show that green entrepreneurship positively contributes to the economic, social, and environmental components of sustainable development during the period 2012–2017. These results demonstrate a measurable indication of sustainable development outcomes, whereby Saudi Arabian institutions align entrepreneurial activities with a positive triple bottom line effect. Accordingly, these findings contribute new evidence to justify government commitment to supporting green entrepreneurship in Saudi Arabia and encourage future domestic policies.

## 1. Introduction

Successful sustainable development meets the needs of the present without compromising those of future generations [1,2,3]. Traditional conceptions of sustainability are typically rooted in the triple bottom line, which includes three major dimensions: social, economic, and environmental [4,5,6,7]. According to Khan [8], various factors related to these three dimensions are used to describe those who tend to exploit sustainable development. The social dimension of sustainability includes factors such as safety, health, and social concerns [4,5]. On the economic front, Svensson and Wagner [7] highlighted factors such as profits and business dynamics. Brocke et al. [9] and Gevrenova [10] stressed the substantial role of green businesses in pursuit of environmentally friendly and sustainable development. The environmental dimension of sustainability covers ecological degradation, carbon labelling, product dematerialisation, and efficiency improvement programmes [7,11]. It has been argued that entrepreneurs, or more specifically, green entrepreneurs, who aim to achieve both business and environmental goals, have a transformative influence on their sectors and play a major role in sustainable development [12,13].

As a driving force for institutional development, entrepreneurship plays a critical role in shaping domestic industries, systems, and networks. Due to systemic forces and institutional variations, however, the degree of influence exerted upon the overarching industry is conditional and heterogeneous across national borders [14,15]. Although analyses of the relationships between institutional factors, entrepreneurship, and development are proliferating, most of the literature remains framed by traditional views such as endogenous growth and Schumpeterian theory [15]. Considering green entrepreneurial activities within the (sustainable) development process requires expanding our perspective, as green entrepreneurs are a part of complex sociotechnical networks and are impacted by other actors, social institutions, policies, and regulations. Zahraie et al. [16] found that green entrepreneurs struggle to break through dominant trends, but regulative support at appropriate moments may help this transition by promoting a vision for collective action. Similar findings were reported by Demirel et al. [17], who suggested that governments play a large role in giving green entrepreneurship legitimacy by awarding contracts, enforcing environmental legislation, or facilitating financing. Yi [18] observed that university-level support of green entrepreneurship fosters an enabling environment for green businesses. Such prior research confirms a positive relationship between green entrepreneurship and green enterprise that is systemically linked to the oversight of governmental support in developing nations. Although scholars in this field have provided evidence supporting the link between environmental entrepreneurship and sustainability in developed economies [16,19], a lack of evidence and academic emphasis on developing countries such as Saudi Arabia raises questions as to the efficacy and transferability of such developmental propositions.

Therefore, the primary aim of this study is to explore the influence of green entrepreneurial activity on sustainable development. Also, the role of entrepreneurship policy is analysed in the context of Saudi Arabia. In accomplishing this aim, institutional economics [20] is used as the theoretical foundation of this research to help us understand the relationship between green entrepreneurship and sustainable development. The utilisation of institutional economics enables us to observe the phenomenon from a different angle, which considers the existence of external factors (e.g., policies) affecting the association of green entrepreneurship with sustainable development. This relationship is tested through panel data models from 13 Saudi Arabian cities during the period from 2012–2017.

Predicting a strong, statistically significant relationship between domestic policies in Saudi Arabia, green entrepreneurship, and the triple bottom line, this study has critically explored time-series evidence from a selective array of multiregional proxies. Using information from the General Authority for Statistics (GAS) in Saudi Arabia, these findings confirm a precipitating relationship between green entrepreneurship and downstream transformation of social, economic, and environmental agendas. Furthermore, this study confirms the role of domestic entrepreneurship policies in supporting and directing entrepreneurial activities toward greener, more sustainable industry outcomes.

Different implications have been derived from this study. First, the influence of green entrepreneurship on sustainable development was analysed by comparing the affective influences of green and nongreen entrepreneurial initiatives on industry outcomes. Second, empirical evidence regarding the social, economic, and environmental advantages of green entrepreneurship were identified, providing a developmental blueprint for improving intra-industry outcomes in future Saudi Arabian ventures. Third, these findings shed light on the differences in approaches towards green entrepreneurship and sustainable development in different regions of Saudi Arabia, highlighting the contagion effect of cross-national knowledge sharing for sustainability in a rapidly developing economy. Finally, the study extends previously available frameworks such as endogenous growth theories and the Schumpeterian theory of entrepreneurship by treating sustainable development as a composite index of economic, social, and environmental dimensions [21]. It also addresses a gap in the existing literature regarding the systemic influence of national regulatory policies on green entrepreneurship and domestic sustainability in Saudi Arabia.

The following section introduces the theoretical framework. Section 3 discusses the conceptual foundations of the literature review and the development of the hypotheses. In Section 4, the methodology and data are explained, and the findings are presented in Section 5. Finally, Section 6 comprises the study’s conclusions, implications, limitations, and suggestions for further research.

## 2. Institutional Economics, Green Entrepreneurship, and Sustainable Development: A Framework for the Saudi Arabian Context

To comprehend the possible mechanisms behind the relationship between green entrepreneurship and sustainable development, this study adopted a paradigm of institutional economics [20,22], a widely utilised theoretical lens for entrepreneurship research on the role of interactions and choices in economic evolution [15,23,24,25,26]. Elaborating on this viewpoint, scholars have explored institutions as antecedents of entrepreneurial activity, as well as their relationships with economic growth [3,15,27,28,29]. Drawing on North [20], institutions are perceived as the source of rules guiding interactions amongst different actors (including firms). Accordingly, the existence of certain institutions creates divergence across regions and countries, as cultures and regulations define different patterns governing production and consumption decisions. For example, North [20] finds differences between Western and Eastern economies, as well as Anglo-Saxon, Scandinavian, German, etc., countries. Although most of the prior research has focused on developed economies [15], there is still a need to understand how institutions work in other places, which might impose barriers to entrepreneurship in different ways [8,30]. Hence, the present study focuses on formal institutions in Saudi Arabia because these more readily inform the decisions of the country’s policymakers.

Saudi Arabia is a strategic and important nation in the Middle East and the world [31,32,33,34]. Saudi Arabia is the largest economy in the Middle East and the richest Arab country in the region [34,35,36]. Petroleum products represent a large majority of exports (77% of total exports in 2019), followed by petrochemical products (around 14% of total exports) [33,34,37]. Machinery and electrical equipment account for the largest share of imports, followed by automobiles, chemical products, and metal products [33,34,38,39]. The policy of large-scale public works undertaken by the authorities, as well as foreign direct investment, means that Saudi Arabia needs to provide governmental supportive policies for green entrepreneurial activities and sustainability. This supports and promotes the aim of reducing the vast overreliance of the economy of Saudi Arabia on oil [40].

Government policies help to establish conditions for boosting environmentally friendly entrepreneurship [41]. The need for development through entrepreneurship has to be balanced with the need to preserve the opportunity for future generations to reach and enjoy a high quality of life and to sustain the environment; this is what the Saudi Arabian government is trying to achieve. The vision for Saudi Arabia in 2030 [42] includes a suite of government-level policies that support economic and social improvements. A particular focus of the Saudi Arabian government and the executive has been to reduce the country’s dependence on oil as one of the major industries and to diversify into other sectors such as clean energy, health, and tourism. Green entrepreneurship and a focus on a holistic approach to economic development that balances people, profit, and planet is thus a cornerstone of Saudi Arabia’s long-term national strategy [43]. Additionally, environmentally sustainable practices develop the social and economic performance of firms in the long term [44].

The policies adopted by Saudi Arabia are consistent with the growing need to address environmental threats and to protect the environment. The work by Yi [18], Alwakid et al. [43], and Ndubisi and Nair [45] suggests that there is a need for companies to adopt a green approach. In developing countries, environmental actions are of prime importance [46]. However, it is not clear whether the actions of the Saudi Arabian government are leading to their intended effects. It is possible that the government either uses resources inefficiently or faces obstacles in implementing environmental policies. For this study, it was critical for additional research related to Saudi Arabia to illuminate whether institutional effects support green entrepreneurship, or whether other factors influence the progression from traditional to sustainable enterprise.

## 3. Literature Review and Hypotheses

### 3.1. Green Entrepreneurship and the Triple Bottom Line

Green entrepreneurship and sustainable development are very closely linked [12]. Previous studies do not always agree on the direction of causality, and the relationship is often viewed as bidirectional with feedback loops [2,47,48]. The problem might come from the conceptualisation itself, as, for example, green entrepreneurship might not only be associated with environmental purposes but also social ones, so there might be an overlap between green and social entrepreneurship [3,43]. In our case, we perceive green entrepreneurial activity as those initiatives focused on solving environmental problems while following rules and regulations oriented to tackling global warming, use of clean energy, recycling standards, etc., [3,13]. By following this approach, we can observe the sustainable entrepreneurship phenomenon as an engine for development shaped by institutional factors [15]. Hence, the natural sequence running from institutions to entrepreneurship and development [15,27,28] helps overcome the bidirectional problem. Yet, this approach is mostly focused on economic outcomes, raising the need to further explore development beyond economic terms. In this regard, Delai and Takahashi [4], Svensson and Wagner [7], and Johnson and Schaltegger [12], amongst others, have tackled this problem through the utilisation of the triple bottom line, which consists of equally weighting those factors involved in sustainability; namely, economic, environmental, and social dimensions. Using this approach suggests that all dimensions are part of a system, which needs balance for perfect functioning [7].

As an example, Rodrigues and Franco [49] developed a composite index of sustainability indicators to assess the affective influences of social, economic, and environmental factors on sustainable development in Portuguese cities. The evidence confirmed measurable developmental effects, including the economic influences on green entrepreneurship, social influences on social cohesion and sustainable lifestyles, and environmental effects on resource management and network efficiency. Similarly, Ukko et al. [50] assessed the affective influence of social, environmental, and economic dimensions on sustainability intentions in shifting SMEs towards improved sustainability commitments. Such findings confirmed the compelling and progressive influence of a triple bottom line on sustainability awareness, priority setting, and enterprise transformation. The following further distils these relationships into their operative dimensions.

#### 3.1.1. Social

The social dimension of sustainability characterises a relationship between entrepreneurship and stakeholder awareness, as businesses target improved responsibility and accountability in safety, health, and social considerations [4,5]. Researchers including Delai and Takahashi [4] and Khan et al. [5] have elaborated on social determinants to explain the relationship between stakeholders and firms through human capital development, job creation, health factors, social recognition, and safety-related issues. Furthermore, Galdeano-Gomez et al. [51] observed the direct correlation between socially responsible enterprise and the exploitation of green entrepreneurship and practices. As a catalyst for organisational change, Ukko et al. [50] determined that social factors such as customer demand and community relationships are antecedents to organisational commitments to sustainable business practices. Despite such positive effects, social sustainability faces a number of challenges, such as balancing social welfare needs against business development and growth [52,53,54]. As a means of progressive social transformation, Cai and Zhou [55] and Jakhar [56] therefore identified social recognition as a major element driving green innovation.

Schaltegger et al. [57] defined green and sustainable entrepreneurship as a process that is attained by solving social and environmental problems through the selection of sustainable market opportunities using innovative techniques and business models. Studying social factors specific to human capital, Del Río et al. [58] and Huang et al. [59] highlighted the notion that human capital development (i.e., training), encourages employees to engage in sustainable methods. Similar evidence captured by Qi et al. [60], and Doran and Ryan [61] supported the argument that commitment to human capital development leads to an increase in information flows that supports corporate innovation, green practices, and entrepreneurial orientation. By embracing what Estrin et al. [62] identify as revised cooperative norms, the collaborative advantages of social entrepreneurship and progressive institutional change stimulate the downstream transformation of inefficient and underperforming systems. However, in transition economies where existing institutions and knowledge systems are weak or underdeveloped, Silajdzic et al. [63] recognised that there is a need for social institutions, individual motives, and forward-thinking orientation to stimulate green entrepreneurship and catalyse institutional change. On the basis of the literature, the following hypothesis was proposed to examine the relationship between green/nongreen entrepreneurship and the social dimension of sustainability:

**Hypothesis 1** **(H1).** *Green entrepreneurship has a higher influence on the social dimension of sustainable development than nongreen entrepreneurship.*

#### 3.1.2. Economic

As both a catalyst for sustainable development and a condition of network efficiency, Zhang et al. [64] observed a direct relationship between green entrepreneurship and economic outcomes. To operationalise such measures, Goodland [65] and Mamede et al. [6] modelled the maintenance of capital in a firm to assess the drivers of green entrepreneurship, highlighting the effects of system and network efficiencies on improved economic propositions. Focusing on specific strategies for improving sustainability, Svensson and Wagner [7] weighed the effects of resource limitations and exhaustible inputs against business innovation and green investments across high-growth, high-innovation channels. Such findings confirm the predictions of Sheth et al. [66], who linked enterprise motivations such as cost reduction and operational efficiency to the sourcing and adoption of greener, higher-performing industry solutions. Similarly, Lee [67] and Lioutas et al. [54] demonstrate the underlying effects of cost-saving motivations on openness to green innovation and entrepreneurship. Beyond such efficiency-based rationalisation, Hojnik and Ruzzier [68], and Horbach et al. [69] demonstrate a systemic connection between negative corporate externalities and emergent cost-saving, impact-reducing strategies that are based upon greener, higher-efficiency eco-innovation. As a catalyst for market innovation and sustainable development, the economic advantages of green entrepreneurship have the ability to stimulate economic activity, increase productivity, maximise competitiveness, and create cutting-edge jobs [70]. Based on the studies covering the economic dimension of sustainability, the following hypothesis is proposed:

**Hypothesis 2** **(H2).** *Green entrepreneurship has a higher influence on the economic dimension of sustainable development than nongreen entrepreneurship.*

#### 3.1.3. Environmental

The acknowledgement and reconciliation of environmental vulnerabilities is a critical antecedent to improving sustainable development [63]. Brocke et al. [9] and Gevrenova [10] reported evidence of the substantial role played by green businesses in the pursuit of environmentally friendly and sustainable development. Suggested measures for promoting a green entrepreneurial spirit included the use of organic products, stringent rules and regulations with regard to emissions and pollution, efficient use of natural resources, and environmentally friendly practices for logistics and supply management [71]. As environmental awareness increases, the pursuit of organisational responsibility and the delivery of greener business solutions is critical to the maintenance of a responsible, eco-friendly reputation and brand identity [62]. Nikolaoua et al. [71] demonstrated that the foundations of green entrepreneurship are developed by those entrepreneurs who are willing to trust innovative technologies and embrace greener, more sustainable products. Based upon these studies regarding the environmental aspect of sustainability, the following hypothesis was proposed:

**Hypothesis 3** **(H3).** *Green entrepreneurship has a higher influence on the environmental dimension of sustainable development than nongreen entrepreneurship.*

### 3.2. The Role of Entrepreneurship Policy in Green Entrepreneurial Activity and Sustainable Development

Three further hypotheses were proffered to test the moderating influence of entrepreneurship policy on the three dimensions of sustainability through the mechanism of green/nongreen entrepreneurial activity. Scholars have explored institutions as antecedents of entrepreneurial activity, as well as and their relationships with economic growth [27,29]. According to Urbano et al. [15], the institutional approach provides a broad insight into understanding how institutions are related to entrepreneurial activity, as well as identifying which institutions are most important in reflecting the entrepreneurship rates that enhance economic growth. From the perspective of institutional economics, formal institutions can adjust their policies much more quickly than informal institutions [22].

These hypotheses stem from the literature on institutional theory, which states that both formal and informal institutions may influence the adoption of sustainable business practices. Governments may foster specific cultural and social norms that correspond to a bidirectional relationship between formal and informal institutions in the framework of institutional economics [22]. Supportive institutional conditions are necessary for the development of green entrepreneurship [72]. This suggests that entrepreneurship policies might moderate the effect of green entrepreneurship by offering additional incentives for socially responsible businesses, which could translate into a positive relationship between sustainability and green entrepreneurship. In contrast, government investments in accelerated economic development or sector-selective growth, such as in the oil and gas industry in Saudi Arabia, have the potential to reduce sustainability and inhibit green entrepreneurial investments [69,70].

To refocus domestic agendas on greener, more sustainable practices, governments also have the capacity to enforce and promote environmentally sound production methods [73] and to influence the policies shaping firm practices and investment objectives [74]. As incentivising measures, domestic entrepreneurship policies can leverage subsidies and target investment sectors in order to systemically increase the commitment to greener, more responsible enterprise [75]. In this regard, the affective influence of entrepreneurial policies could affect economic, social, and environmental issues.

Institutional theory predicts that through shifting policy measures and government support, institutional changes have the potential to shift social, cultural, and economic values towards improved sustainability [20]. As a catalyst for green entrepreneurship, government commitments to supportive and responsible institutional policies have the potential to catalyse change and encourage greater investment in innovative and responsible practices [75]. However, governments may prioritise more immediate social or economic problems over environmental concerns and therefore adjust entrepreneurship policies to tackle inequality, unemployment, poverty, and infrastructure deficiencies rather than pursue sustainable development [72]. The net moderating impact of entrepreneurship policy on the environmental aspect of sustainable development may then be ambiguous. A consequence of this approach concerns the government’s abovementioned capacity to enforce and promote environmentally sound production methods [73]. On the basis of institutional theory, three additional hypotheses were therefore proposed:

**Hypothesis 4A** **(H4A).** *Entrepreneurship policy has a positive moderating influence on the relationship between green entrepreneurship and the social dimension of sustainable development.*

**Hypothesis 4B** **(H4B).** *Entrepreneurship policy has a positive moderating influence on the relationship between green entrepreneurship and the economic dimension of sustainable development.*

**Hypothesis 4C** **(H4C).** *Entrepreneurship policy has a positive moderating influence on the relationship between green entrepreneurship and the environmental dimension of sustainable development.*

**Figure 1:**
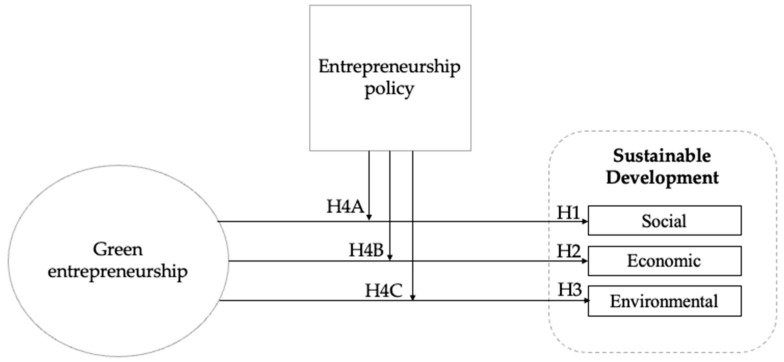
Conceptual model.

Figure 1 portrays our conceptual model.

## 4. Materials and Methods

### 4.1. Data and Variables

This section describes the data, sample, and methodology used in the present study, which drew on the reports from the General Authority for Statistics (GAS) and annual reports of the General Authority for Meteorology and Environmental Protection (GAMEP). Regional data for 13 cities in Saudi Arabia for the period 2012–2017 were extracted, and the cities were used as proxies for the regions. The two main independent variables in the study were green and nongreen entrepreneurship in Saudi Arabia. Entrepreneurship policy constituted the third independent variable. The dependent variable was sustainable development. Data on this variable were not readily accessible and therefore had to be constructed as a composite index based on the available information. Appendix A contains a full table detailing the dependent, independent, and control variables.

#### 4.1.1. Dependent Variables

The present study subdivided sustainable development into economic, social, and environmental components, mirroring the prior work conducted by Potluri and Phani [21], who explored green entrepreneurship using the resource-based view (RBV). Following Secundo et al. [76], the social dimension of sustainable development consisted of the following variables: (a) healthcare as a percentage of total government expenditure in the health and social development sector; (b) social policy—social investment in quality of life (i.e., total spending on development); (c) education as a percentage of total government expenditure on education; and (d) security as a percentage of total government expenditure on security and regional administration.

The social dimension of sustainable development was represented by a composite of several aspects, taking inspiration from the approach of Lee et al. [67] and Potluri and Phani [21], who observed that that higher levels of education and healthcare play a major role in sustainability. They also suggested that the achievement of sustainable development requires effective responses to a wide range of social issues, including inequality, insecurity, and conflict. This justifies government expenditure in various social sectors and represents the social dimension of sustainable development.

The economic dimension of sustainable development was represented by a composite of three aspects, taking inspiration from the composite approach of Potluri and Phani [21] to source core variables, and from the arguments of Lee et al. [67], which predict the direct intersection between sustainability and finance in the modern world. Accordingly, the economic dimension of sustainable development in the present study included: (a) Saudi Arabia’s employment and unemployment rates (using data gathered from the General Authority for Statistics); (b) the level of financial development as measured by the density of banks, which were an indicator of economic growth driven by demand; and (c) the level of entrepreneurship and competition, as measured by the proportion of small businesses within the market.

These factors were consistent with numerous studies confirming the existence of a relationship between the presence of small firms and levels of entrepreneurial activity. For example, Qi et al. [60] and Goldstein [77] claimed that sustainability cannot be analysed in isolation from financial inclusion and financial sector development. Eustachio et al. [78] studied global sustainability goals and concluded that economic activity and employment were essential elements of sustainability. The present study’s inclusion of small businesses followed the strategy of Cantele and Zardini [79], who found that small enterprises gained significant competitive advantage through green entrepreneurship.

The environmental dimension of sustainable development was measured by several variables, including (a) waste management and (b) recycling, based on empirical evidence of how the use of recycling and waste reduction helped to achieve sustainable production [80]. Preservation of the environment in cities and in rural regions has been used frequently as a factor in previous empirical research [80,81,82]. Additional variables are (c) development assistance to conserve biological diversity and (d) the agricultural trend index (GAS data). All variables were rescaled to obtain comparable value ranges.

Appendix B provides a summary of the factor analysis of the economic, social, and environmental components of sustainable development. Typically employed as a reductive instrument, PCA distils variance to its maximal and minimal factors, weighting variations to determine a discriminate representation of each observation vector [83]. The components of sustainable development were taken as the first principal components of the corresponding decomposition. The Kaiser–Meyer–Olkin Index was at least 0.75 for all three components of sustainable development, indicating that it was appropriate to use factor analysis to describe the data. Indicating a robust statistical representation, the weight of variance demonstrates a direct, significant relationship between key components and the underlying effects on sustainable development.

#### 4.1.2. Independent Variables

The core independent variables were measures of green and nongreen entrepreneurship. In Saudi Arabia, no database exists for green entrepreneurship; therefore, a proxy measure was adopted. The First Voluntary National Review [84] determined whether Saudi Arabian firms had adhered to the standards of green entrepreneurship. This evaluation was based on the parameters set by the United Nations, which has called for the development and growth of businesses that meet sustainability goals. To measure these variables, we considered the number of firms that had adopted an environmentally sustainable business model as a proxy for green entrepreneurship, and the number of firms with high pollution rates (e.g., tonnes of carbon emissions) based on annual reports from the GAMEP as a proxy for nongreen entrepreneurship.

Entrepreneurship policy constituted another important independent variable in the present study. This independent variable expanded upon the findings of Obaji and Olugu [85] by exploring the moderating influence of entrepreneurship policy on the relationships between entrepreneurship and various dimensions of sustainable development. Entrepreneurship policy is understood as the set of incentives and government procedures that facilitates the entrepreneurial process of establishing a company. Shuo [73] explained how governments apply different mechanisms, such as subsidies, tax incentives, and government procurement guidelines, which enhance the economy’s capacity to support entrepreneurial activity and affect entrepreneurs directly. All variables were rescaled to obtain comparable value ranges on a five-point Likert-style scale: 1 = very low or none, 2 = low or minor, 3 = moderate or significant, 4 = high, and 5 = very high. Each indicator was measured in percentage. The Likert scale relied upon a range of percentages to group the outputs: (1) low percentages between zero and 20%, (2) low or minor percentages between 21% and 40%, (3) moderate or significant percentages between 41% and 60%, (4) high percentages between 60% and 80%, and (5) very high percentages between 80% and 100%.

#### 4.1.3. Control Variables

Other variables were also included in the model to control for additional factors that might help to explain sustainable development. We controlled for Saudi Arabia’s national annual growth rate, which represented the value of the country’s resources and which is increasingly sensitive to competitive forces in world markets. Environmental issues are sensitive to world markets because they shape the potential for economic growth by conditioning survival. In Saudi Arabia, the unsustainable use of resources is an important issue that is perpetuated by domestic dependence upon the oil and gas industry [86]. The annual growth rate was extracted from the annual reports of the General Authority for Statistics (GAfS) (2012–2017). Values for the annual growth rate were drawn from the five-year average for each city.

Another variable, environmental consciousness, was measured as the percentage of natural resources that were maintained at an appropriate level. This variable represented the reduction in the use of natural resources relative to output, that is, the extent to which a city was balanced in its use of natural resources [87]. According to Alwakid et al. [43], environmental consciousness is positively associated with green entrepreneurship in Saudi Arabia. We controlled for the population of the area studied, since green entrepreneurship aims to minimise threats that may occur because of a decrease in natural resources, such as an increase in population growth [88,89]. The data for this control variable were again extracted from the annual reports of the GAfS. The variable’s value was population size, which increased in each area during the five-year study. The size of a city (which was included as a control variable) may affect the availability of natural resources and also its rate of natural resource depletion; a larger city leads to a greater demand for natural resources [86].

The level of education in each city was included as a control variable. Governments aim to improve access to high-quality education, which may be required for the achievement of sustainable development at all levels and in all social contexts [49]. Effective policies can transform society by reorientating the education system and helping individuals to develop the knowledge, skills, values, and behaviours needed for sustainable development [90]. This variable was measured by the percentage of people with a postgraduate degree in each city. According to Abdul [91], an increase in the number of postgraduate students is of the utmost importance to entrepreneurship. The average number of beneficiaries of basic services (e.g., water and electricity utilities) and economic activity—as measured by per capita growth in total output—was included as an additional control variable.

This study also controlled for the preservation of the environment in the agricultural and municipal sectors, namely through temporal orientation, which was defined as the rate at which public and private organisations adopted environmental measures in each city. According to Alwakid et al. [43], temporal orientation is positively associated with green entrepreneurship in Saudi Arabia. Entrepreneurs operating in environments of high temporal orientation often need to compete with other firms by taking advantage of the dynamic market conditions to create novel products or services, thus addressing emerging environmental needs [92]. The final control variable was innovation policy. This is a relatively new concern for policymakers [93]. Mohnen and Röller [94] noted that innovation policy encompassed a range of policies that encouraged firms to create and offer new products and services. The values for this particular variable were based on a five-point Likert-style scale: 1 = very low to 5 = very high. Appendix A provides further details about the variables.

The entrepreneurial orientation of the firms in the dataset was determined either by their age (new) and/or size (small) measured by turnover at the time of the data collection. Kücher et al. [95] regarded any firm under the age of three to be entrepreneurial in nature. Beyond that period, the firm is considered typically to have moved into a secondary phase of maturity [96]; as both regional and global studies on the survival rates of small firms have illustrated, failure is most likely to occur within these initial three years. Firm size was also treated as a proxy for entrepreneurial activity. Revilla et al. [97] showed that smaller firms retain characteristics of entrepreneurship and entrepreneurial orientation even as they mature. This is evidenced by agility, responsiveness, and adaptability.

### 4.2. Modelling Approach

Fixed effects (FE) models were used to test whether green and nongreen entrepreneurship influenced sustainable development and to test the moderating influence of entrepreneurship policy on the relationship between green entrepreneurship and various dimensions of sustainable development. Equation (1) specifies the overall FE model.

(1) $$\begin{array}{c} SD_{it}=\alpha +\beta_{1}GR_{it}+\beta_{2}NonGR_{it}+\beta_{3}GR_{it}\times EntP_{it}+\beta_{4}NonGR_{it}\times EntP_{it}\\ +\beta_{5}EntP_{it}+\gamma Controls_{it}+\varepsilon_{it} \end{array}$$

where SD is one of the three components of sustainable development, GR and NonGR are green and nongreen entrepreneurship, EntP is entrepreneurship policy, and Controls is the vector of the control variables. Each variable was normalised by its standard deviation and transformed using natural logarithms to improve the fit of the linear model.

The use of the FE technique allowed observation of the time effects in a cross-regional approach [98,99]. Panel data are also better able to measure and identify effects not detectable simply in pure cross-section or pure time-series data [98]. This study focused only on the fixed effects, since utilising the full fixed model and carrying out the selection on the random effects within it resulted in additional noise, which stemmed from unnecessary fixed effects [98]. Accordingly, it was possible to capture changes in Saudi Arabian cities over time, which have different economic, geographical, and social characteristics, all of them observable through fixed effects.

A city-level analysis provided a more detailed exploration of entrepreneurship trends, both within and between states, as these can vary significantly [62]. In addition, different cities may have increased the level and regularity of observations, leading to higher levels of confirmed and verified results. Considering different cities in an array of locations allowed the opportunity to evaluate any significant influence, and the panel data technique modelled time effects using a cross-regional approach [63].

## 5. Results

The key descriptive statistics for the variables are shown in Table 1. Economic factors varied from −2.247–3.484 (M = 0.000, SD = 1.653). Social factors ranged from −2.526–4.044 (M = 0.000, SD = 1.578). Environmental factors ranged from −2.566–2.992 (M = 0.000, SD =1.627).

Pearson’s correlation revealed that some of the variables had significant positive relationships and others insignificant relationships. For example, environmental factors showed a strong correlation with green entrepreneurship (r = 0.916), whereas there was a moderate correlation between social factors and nongreen entrepreneurship (r = 0.643). Table 2 shows that both green entrepreneurship and nongreen entrepreneurship were highly correlated with the components of sustainable development. The correlation between independent variables was moderate to low, suggesting that there were no multicollinearity problems in the sample. Entrepreneurship policy did not appear to be correlated to the components of sustainable development.

Table 3 illustrates a synthesis of the key results for all of the panel data models with fixed effects evaluating social, economic, and environmental dependent variables (see Appendix C, Appendix D and Appendix E). Only the controlled variables were included in models 1, 4, and 7. The other three models (2, 5, and 8) were then set, each with one predictor representing each hypothesis. Finally, additional models (3, 6, and 9), which included all predictors (i.e., independent variables, controls, and the interaction terms) were explored. Throughout this empirical strategy, tests were performed to assess whether different linear combinations created different results or whether a robust specification was found; the full tables are presented in Appendix C, Appendix D and Appendix E.

Regarding the hypothesis testing, there was a positive association between green entrepreneurship, nongreen entrepreneurship, and sustainable development in different regions of Saudi Arabia, so H1 was not rejected. These findings confirmed that green entrepreneurship had a significant positive effect on the dependent variable in the full model (2.179, *p* < 0.01), whereas nongreen entrepreneurship had a nonsignificant effect. H2 argued that green entrepreneurship had a higher influence on the economic dimension of sustainable development than nongreen entrepreneurship. The evidence indicated that green entrepreneurship was positively related to the economic dimension. As with Pozdniakova [70], this study demonstrated that green entrepreneurship had a significant positive influence on the dependent variable in the full model (1.220, *p* < 0.05), whereas nongreen entrepreneurship had no significant impact on economic factors; this was consistent with H2.

The third hypothesis, H3, suggested that green entrepreneurship had a higher influence on the environmental dimension of sustainable development than nongreen entrepreneurship. Green entrepreneurship was positively associated with the environmental dimension, so H3 was fully supported. This was consistent with the empirical findings of Svensson and Wagner [7]. In addition, the results indicate that green entrepreneurship had a significant positive influence on the dependent variable in the full model (1.117, *p* < 0.01), whereas nongreen entrepreneurship had a non-significant negative influence. Thus, only green entrepreneurship appeared to boost the environmental component of sustainable development.

Concerning interactions, H4A suggested that entrepreneurship policy has a positive moderating influence on the relationship between green entrepreneurship and the social dimension of sustainable development. The interaction term for green entrepreneurship was not statistically significant; therefore, green entrepreneurship was found to have a similar influence on social factors regardless of entrepreneurship policy, so H4A was rejected. As Prasetyo and Kistani [25] suggested, the link between social entrepreneurship and social capital under conditions of low government activism and rising domestic competitiveness might explain the lack of influence of entrepreneurship policy on the relationship between green entrepreneurial activity and the social dimension of sustainable development.

H4B posited that entrepreneurship policy has a positive moderating influence on the relationship between green entrepreneurship and the economic dimension of sustainable development. Despite green entrepreneurship having a significant positive influence on the dependent variable in the full model, the interaction term for green entrepreneurship was negative and significant at the 0.10 level, suggesting that the influence of green entrepreneurship on economic factors decreased with the quality of entrepreneurship policy. This contradicted H4B. This might be explained by the type of incentives offered in the entrepreneurship policy, which might encourage other types of firms with less environmental consciousness [75].

We suggested in H4C that entrepreneurship policy has a positive moderating influence on the relationship between green entrepreneurship and the environmental dimension of sustainable development. Even though green entrepreneurship positively explained the dependent variable in the full model, the interaction term for green entrepreneurship was not significant at the 0.10 level. This might indicate that the positive impact of green entrepreneurship on environmental factors did not depend on the quality of entrepreneurship policy, thus contradicting H4C. Thus, only green entrepreneurship appeared to boost the environmental component of sustainable development, which is consistent with the extant literature [7,63].

In summary, a comparison of H1, H2, and H3 showed strong significant relationships between proactive green entrepreneurship and social, economic, and environmental outcomes, but the data suggested that nongreen entrepreneurship had a non-significant effect. It was therefore concluded that, overall, there was a statistically significant relationship between green entrepreneurship and sustainable development outcomes.

To affirm these results, Cronbach’s alpha was calculated as a measure of internal reliability of the Likert-scored elements of the research instrument (innovation and environment policies). Cronbach’s alpha, denoted by α and calculated by the Equation (2)

(2) $$\;a=\frac{k}{k-1}(1-\frac{\sum Vi}{Vt})$$

is a measure of internal reliability and consistency. It contained the following elements in the present instance: a count of the items (2), a count of the sum of the items (343), and a sum of the variance of the items (16.35). Unfortunately, there appeared to be limited internal reliability. Possible explanations for this include gaps in the data and uncertainty over their interpretations in different regions. Further studies would therefore be necessary to determine causality, as has been previously discussed.

## 6. Conclusions

Prior research regarding the association between green entrepreneurship, nongreen entrepreneurship, and sustainable development in Saudi Arabia is limited. This study has illuminated a positive, regionally heterogeneous relationship between green and nongreen entrepreneurship and sustainable development. In particular, green entrepreneurship had a stronger influence than nongreen entrepreneurship on all the dimensions of sustainable development. Our results are consistent with previous studies that have shown tight links and interrelations between green entrepreneurship and sustainable development [2,47,48]. The findings also correspond with more recent work that has recognised the bidirectional nature of green entrepreneurship and sustainable development in urban contexts [50,52].

By contrast, the results on the moderating influence of entrepreneurship policy were mixed. None of the three corresponding hypotheses were confirmed, indicating that a domestic policy does not have a positive moderating influence on the relationship between green entrepreneurship and sustainable development. Interestingly, a negative moderating influence was found for the economic component of sustainable development. This could suggest that existing entrepreneurship policies in Saudi Arabia may impair the positive influence of green businesses on the country’s economic sustainability. When viewed through the lens of institutional economics, the results can be considered consistent with the work of Urbano et al. [15], in that business was hindered by high levels of corruption and weak property rights. As North [22] reminds, it is possible for institutional support to be focused on economic growth. This could have a negative moderating influence and might explain the outcome of H4A–H4C. Yet, our results might support a debate offered by Yi [18], who emphasised the role of external institutional support in translating green entrepreneurship intentions into actions.

The lack of a moderating influence of entrepreneurship policy on the link between green entrepreneurship and economic and environmental factors might reflect the degree of sustainability awareness amongst both producers and consumers [52]. Alternatively, a non-significant effect represents a net zero impact of positive and negative externalities of governmental policies. The Saudi Arabian government may not be providing adequate instruments for green entrepreneurs to deal with existing risks and uncertainties, which will impair sustainable development [41,74]. Hence, our analysis may serve to derive theoretical and policy implications.

### 6.1. Theoretical Implications

Green entrepreneurship is a novel field of research, so further exploration is needed with respect to the role of entrepreneurial activity as a means of sustaining the environment and ecosystems, while advancing both economic and non-economic gains for investors and society in general [89,93]. Research into the influence of formal institutions on certain outcomes in green entrepreneurship should be founded on theory. The present study has advanced knowledge in the field, in that it has tested existing theoretical propositions robustly and comprehensively and has confirmed the role of green entrepreneurship in sustainable development. We also consider that our empirical findings may better guide scholars studying Saudi Arabia to help entrepreneurs to become more aware of sustainability policies. It may also serve to encourage the advertising of outcomes related to sustainability as a way of increasing the legitimacy of policies and generating the support of entrepreneurs.

In addition, it builds on the work of Potluri and Phani [21] and Huang et al. [59] by pointing to the impossibility of parsing sustainability and the development of the financial sector development in the current context. Our results serve to call the attention of those scholars analysing entrepreneurship from an institutional perspective [15]. Accordingly, we extend the notion of entrepreneurship with environmental purposes as an antecedent of outcomes beyond economic terms. This implies that our evidence of Saudi Arabia can exemplify the conceptual structure, which suggests that institutions determine green entrepreneurial activity needed for social, economic, and environmental development [100].

### 6.2. Policy Implications

The findings of the present study are consistent with Khan [8], who claimed there were not enough associations and institutions in Saudi Arabia lobbying for sustainable business practices. Therefore, policies designed by the relevant Saudi authorities might not be taking into account important entrepreneurship networks. This could reduce the number of opportunities for new businesses and impair the development of green entrepreneurship in the country [13,17]. Saudi Arabia only has a small number of business incubators [8], which may limit the availability of value-added assistance for green entrepreneurs [85].

Government can affect the engagement of entrepreneurs by helping them in their understandings and applications of sustainable development policies. There are other important implications for the analysis of formal institutions [20,22]. For example, if green entrepreneurs have strong bonds with governments, they feel valued by local and national entities, so their opinions and actions are positively considered in sustainable developmental processes. Government support for green entrepreneurship allows for a more sustainable environment, and can be the first step toward a more environmentally conscious society and for the conservation of resources for future generations. The government of Saudi Arabia, in particular, should continue to promote such policies.

### 6.3. Limitations and Future Research

Central to the indicators of sustainability, the current study applied an array of prior models and proxy dimensions to assess the particular traits of the Saudi Arabian social, economic, and environmental systems. Furthermore, institutional conditions were measured in relation to incongruous incentivisation schemes and scalar comparisons of cross-geographic indicators of entrepreneurship. These approaches, although yielding a diversified quantitative model, resulted in several critical limitations that have skewed and diluted the efficacy of these findings. For example, H4A–H4C were rejected due to the inconsistent effects of policy measures on green entrepreneurship. This limitation, however, is likely linked to the proxy indicators, a constraint that will be reconciled in future research where government performativity is used to track progress towards Vision 2030 sustainability objectives. Another example of a proxy-based limitation in this study was the assumption of relational causality between input–output variables. The measure of nongreen entrepreneurship, for example, was based upon an assumption of a direct correlation between high pollution rates and nongreen business activities. This indicator implies distinction between green and nongreen businesses on the basis of carbon footprint, but does not control independently for size or industry of enterprise. It is recommended that future researchers test the relationship between green entrepreneurship and sustainable development by using different proxies for social, environmental, and economic aspects to ensure confidence in the policy application of their findings. By weighting the effects of specific policy measures in developing nations such as Saudi Arabia against sustainability indicators over longitudinal models of green entrepreneurship or domestic sustainability, it is predicted that future evidence will confirm the affective influence of targeting strategies on social, economic, and environmental outcomes. Finally, future research could carry out more cross-sectional and longer-term analyses by investigating evidence from other developing countries within the GCC region and by extending the present study’s six-year time frame.

## Figures and Tables

**Table 1:** Summary statistics.

Variable	N	Mean	Std. Dev.	Min	Max
Dependent					
Economic Factors	78	0.000	1.653	−2.247	3.484
Social Factors	78	0.000	1.578	−2.526	4.044
Environmental Factors	78	0.000	1.627	−2.566	2.992
Independent					
Green Entrepreneurship	78	56,204.81	69,147.11	1025	253,653
Nongreen Entrepreneurship	78	80,906.64	74,476.37	9241	254,032
Controls					
Resources	78	3.475	1.291	1.402	7.421
Population	78	2,232,516	2,737,204	139,114.2	14,200,000
Education	78	6.408	1.875	3.262	11.181
City Size	78	144,677.1	137,752	5,287.588	769,082.3
Economic Activity	78	0.255	1.154	−4.419	2.196
Environmental Preservation	78	30.090	17.000	6	87
Basic Services	78	82.723	15.388	46.442	100.000
Environmental Consciousness	39	6.182	3.081	2.108	15.536
Innovation Policy	52	2.085	0.954	1	5
Temporal Orientation	39	59.347	22.779	25.721	135.861
Interaction Variables					
Entrepreneurship Policy	52	1.783	0.819	1	5

**Table 2:** Correlation matrix.

	VARIABLE	1	2	3	4	5	6	7	8
1	Economic Factors	1.000							
2	Social Factors	0.836	1.000						
3	Environmental Factors	0.938	0.862	1.000					
4	Green Entrepreneurship	0.943	**0.811**	0.916	1.000				
5	Nongreen Entrepreneurship	0.795	0.643	0.768	0.893	1.000			
6	Resources	−0.074	**−0.072**	**−0.096**	−0.116	−0.096	1.000		
7	Population	0.602	0.398	0.570	0.606	0.690	0.134	1.000	
8	Education	0.540	0.407	0.481	0.582	0.531	0.126	0.486	1.000
9	City Size	0.005	−0.011	0.045	**−0.004**	0.041	0.129	0.154	−0.065
10	Economic Activity	−0.080	−0.090	**−0.068**	−0.083	−0.119	−0.139	−0.146	−0.187
11	Environmental Preservation	−0.013	−0.037	−0.012	**−0.001**	−0.018	−0.141	0.023	0.000
12	Basic Services	0.171	0.087	0.163	0.077	0.083	0.143	0.179	−0.054
13	Environmental Consciousness	−0.119	0.075	0.041	**−0.087**	**−0.053**	0.035	0.062	−0.102
14	Temporal Orientation	−0.108	−0.131	−0.130	−0.163	−0.229	−0.147	**−0.069**	−0.205
15	Innovation Policy	0.189	0.279	0.198	0.169	0.140	−0.045	0.150	0.138
16	Entrepreneurship Policy	0.054	0.018	**−0.007**	0.028	0.020	0.028	**−0.001**	−0.182
9	City Size	1.000							
10	Economic Activity	0.136	1.000						
11	Environmental Preservation	−0.037	**0.073**	1.000					
12	Basic Services	0.210	0.040	−0.174	1.000				
13	Environmental Consciousness	−0.193	−0.229	−0.212	0.353	1.000			
14	Temporal Orientation	**−0.013**	0.161	0.371	0.021	**−0.036**	1.000		
15	Innovation Policy	0.160	−0.177	−0.214	0.058	0.083	−0.189	1.000	
16	Entrepreneurship Policy	−0.103	**−0.064**	−0.355	0.023	−0.130	−0.167	0.068	1.000

Correlations in bold are significant at *p* < 0.01.

**Table 3:** Social, economic, and environmental factors.

	Social Factors			Economic Factors			Environmental Factors			
Main independent variables										
Green Entrepreneurship	0.910 ***	2.182 ***	2.179 ***	1.077 ***	1.185 *	1.220 **		1.066 ***	1.115 ***	1.117 ***
	(0.118)	(0.609)	(0.666)	(0.142)	(0.560)	(0.431)		(0.162)	(0.322)	(0.328)
Nongreen Entrepreneurship	0.208	−6.294	−6.228	−2.053	−9.174	−10.407		−0.526	−5.821	−5.848
	(2.318)	(5.077)	(5.342)	(1.179)	(5.704)	(6.070)		(2.687)	(6.028)	(5.996)
Interaction terms										
Green x			−0.011			−0.739 *				0.004
Entrepreneurship Policy			(0.362)			(0.346)				(0.229)
Nongreen x			0.007			1.071 **				−0.002
Entrepreneurship Policy			(0.487)			(0.483)				(0.320)
Controls										
Entrepreneurship		−0.064	−0.079		−0.119	−0.796 **			0.090	0.096
Policy		(0.154)	(0.401)		(0.185)	(0.281)			(0.117)	(0.224)
									
Resources	0.101	0.436 **	0.438 **	0.008	−0.002	−0.011		0.036	0.121	0.121
	(0.280)	(0.182)	(0.188)	(0.131)	(0.173)	(0.186)		(0.110)	(0.135)	(0.148)
Population	−0.041	−0.398 *	−0.396	0.146	0.394	0.434 **		0.015	0.079	0.078
	(0.233)	(0.217)	(0.241)	(0.085)	(0.254)	(0.191)		(0.073)	(0.191)	(0.196)
City Size	−0.249	−0.370	−0.371	0.119	0.098	0.272		−0.036	0.355 **	0.356 *
	(0.256)	(0.233)	(0.275)	(0.116)	(0.226)	(0.204)		(0.118)	(0.147)	(0.168)
Education	−0.023	−0.404 *	−0.398	0.028	−0.693	−0.625		0.319 **	0.356	0.353
	(0.294)	(0.222)	(0.235)	(0.133)	(0.406)	(0.452)		(0.127)	(0.315)	(0.326)
Economic Activity	−0.040	0.030	0.028	−0.013	0.003	−0.028		0.029	0.029	0.029
	(0.084)	(0.081)	(0.105)	(0.063)	(0.065)	(0.059)		(0.032)	(0.032)	(0.034)
Environmental Preservation	−0.133	0.320 **	0.320 *	−0.050	−0.072	−0.024		0.033	−0.004	−0.004
	(0.123)	(0.145)	(0.161)	(0.062)	(0.089)	(0.111)		(0.081)	(0.091)	(0.099)
Basic Services	−0.640	−0.457	−0.467	0.402	1.055	1.142		−0.321	−0.229	−0.225
	(0.685)	(0.663)	(0.789)	(0.310)	(0.699)	(0.674)		(0.223)	(0.540)	(0.575)
Environmental		−0.228	−0.225		−0.483 *	−0.253			0.270	0.269
Consciousness		(0.167)	(0.200)		(0.230)	(0.303)			(0.321)	(0.349)
Temporal Orientation		−0.183	−0.187		−0.063	−0.185			−0.005	−0.003
		(0.346)	(0.384)		(0.244)	(0.188)			(0.167)	(0.177)
Innovation Policy		0.406	0.409		−0.105	−0.194 **			−0.034	−0.036
		(0.241)	(0.271)		(0.144)	(0.088)			(0.091)	(0.109)
Constant	2.159	1.301	1.329	0.078	−1.871	−2.348		1.256	−0.725	−0.737
	(1.767)	(1.678)	(1.800)	(0.687)	(2.230)	(1.952)		(0.993)	(1.546)	(1.536)
N	78	39	39	78	39	39		78	39	39
R^2^ within	0.476	0.789	0.789	0.807	0.447	0.625		0.823	0.732	0.732
R^2^ between	0.709	0.389	0.387	0.075	0.714	0.724		0.860	0.636	0.637
R^2^ overall	0.644	0.305	0.302	0.031	0.676	0.684		0.842	0.589	0.590

* *p* < 0.10, ** *p* < 0.05, *** *p* < 0.01. Robust standard errors in parentheses.

## Data Availability

Data are available under request to the authors.

## Appendix A

**Table A1:** Descriptions of variables.

Variable	Definition	Data Source	
Dependent variable:			
Sustainable development:			
Economic components	The economic dimension of sustainable development included: Growth in employment (employment rate) Number of small business as a proportion of the broader economy Density of banks (number of bank branches)	Annual reports of the General Authority for Meteorology and Environmental Protection	
Social components	The social dimension of sustainable development included: Percentage of total government expenditure in the health and social development sector Social investment in quality of life (total spending on development) Percentage of total government expenditure on education Percentage of total government expenditure on security and regional administration		
Environmental components	The environmental dimension of sustainable development included: Waste management Recycling rate Development assistance to conserve biological diversity The agricultural trend index		
Independent variables			
Green entrepreneurship	Number of firms considering the environment in the city	Annual reports of the General Authority for Meteorology and Environmental Protection	
Nongreen entrepreneurship	Number of firms with high pollution rates		
Interaction variable			
Entrepreneurship policy	Set of incentives and government procedures that facilitate the establishment of entrepreneurial projects. Measured on a 5-point scale (1 = very low, 5 = very high)	Annual reports of the Authority for Meteorology and Environmental Protection	
Control variables			
Population	Number of inhabitants per region	Annual reports of the General Authority for Statistics in Saudi Arabia	
Size	Area of each city (km^2^)		
Annual growth rate (resources)	Annual growth rate for each city		
Environmental consciousness	Percentage of natural resources that were maintained at an appropriate level		
Level of education	Percentage of people with postgraduate degrees		
Basic services	Average number of beneficiaries of basic services		
Economic activity	Annual growth rate per capita		
Environmental preservation	Preservation and protection of the environment (measured as a percentage of spending on municipal services)		
Temporal orientation	Rate of adoption of environmental measures by public and private organisations in each city		
Innovation policy	Interface between technological development policy, research, and industrial policy, which aims to create a framework for bringing new ideas to the market. Measured on a 5-point scale (1 = very low, 5 = very high)		

## Appendix B

**Table A2:** Summarised factor analysis (PCA).

Variable	Comp1	Comp2	Comp3	Comp4
	Economic factors			
Employment	0.581	−0.490	0.650	
Unemployment	0.586	−0.302	−0.752	
Density of banks	0.565	0.818	0.112	
Proportion	0.910	0.064	0.026	
KMO (Total)	0.750			
	Social factors			
Education	0.546	0.008	−0.216	−0.809
Health	0.472	0.787	−0.152	0.367
Security	0.488	−0.176	0.849	0.101
Quality of Life	0.491	−0.591	−0.457	0.448
Proportion	0.622	0.156	0.133	0.089
KMO (Total)	0.767			
	Environmental factors			
Recycling	0.583	−0.178	−0.793	
Development	0.572	0.783	0.245	
Agricultural Trend	0.577	−0.596	0.558	
Proportion	0.882	0.068	0.050	
KMO (Total)	0.764			

## Appendix C

**Table A3:** Regression analysis (DV = social factors).

	1	2	3	4	5	6	7	8	9
Main independent variables									
Green Entrepreneurship			0.883 ***		2.254 ***		0.910***	2.182 ***	2.179 ***
			(0.112)		(0.617)		(0.118)	(0.609)	(0.666)
Nongreen Entrepreneurship				−4.613		−11.274 *	0.208	−6.294	−6.228
				(4.796)		(6.264)	(2.318)	(5.077)	(5.342)
Interaction terms									
Green x									−0.011
Entrepreneurship Policy									(0.362)
Nongreen x									0.007
Entrepreneurship Policy									(0.487)
Controls									
Entrepreneurship		0.286 *			−0.012	0.176		−0.064	−0.079
Policy		(0.132)			(0.167)	(0.110)		(0.154)	(0.401)
Resources	−0.083	0.253			0.406 *	0.316	0.101	0.436 **	0.438 **
	(0.341)	(0.327)			(0.196)	(0.293)	(0.280)	(0.182)	(0.188)
Population	−0.018	−0.872 *			−0.512 **	−0.647	−0.041	−0.398 *	−0.396
	(0.243)	(0.435)			(0.221)	(0.374)	(0.233)	(0.217)	(0.241)
City Size	0.037	−0.455			−0.379	−0.433	−0.249	−0.370	−0.371
	(0.313)	(0.472)			(0.247)	(0.486)	(0.256)	(0.233)	(0.275)
Education	−0.163	0.344			−0.163	−0.116	−0.023	−0.404 *	−0.398
	(0.325)	(0.522)			(0.220)	(0.440)	(0.294)	(0.222)	(0.235)
Economic Activity	0.002	−0.079			0.045	−0.100	−0.040	0.030	0.028
	(0.079)	(0.098)			(0.073)	(0.111)	(0.084)	(0.081)	(0.105)
Environmental Preservation	−0.155	0.310 *			0.307 *	0.334 **	−0.133	0.320 **	0.320 *
	(0.154)	(0.171)			(0.144)	(0.152)	(0.123)	(0.145)	(0.161)
Basic Services	−0.731	−1.688 *			−0.480	−1.577	−0.640	−0.457	−0.467
	(0.837)	(0.912)			(0.713)	(0.912)	(0.685)	(0.663)	(0.789)
Environmental		0.340			−0.099	0.085		−0.228	−0.225
Consciousness		(0.311)			(0.187)	(0.276)		(0.167)	(0.200)
Temporal Orientation		0.464			−0.104	0.291		−0.183	−0.187
		(0.566)			(0.324)	(0.566)		(0.346)	(0.384)
Innovation Policy		0.523 *			0.373	0.574 **		0.406	0.409
		(0.242)			(0.245)	(0.203)		(0.241)	(0.271)
Constant	1.548	0.353	1.124 ***	−1.745	3.179 **	−2.851	2.159	1.301	1.329
	(1.496)	(2.120)	(0.142)	(1.815)	(1.397)	(2.890)	(1.767)	(1.678)	(1.800)
N	78	39	78	78	39	39	78	39	39
R^2^ within	0.043	0.485	0.435	0.047	0.778	0.522	0.476	0.789	0.789
R^2^ between	0.051	0.478	0.741	0.575	0.755	0.597	0.709	0.389	0.387
R^2^ overall	0.000	0.311	0.658	0.414	0.735	0.525	0.644	0.305	0.302

* *p* < 0.10, ** *p* < 0.05, *** *p* < 0.01.

## Appendix D

**Table A4:** Regression analysis (DV = economic factors).

	1		2	3	4	5	6	7	8	9
Main independent variables										
Green Entrepreneurship				1.117 ***		1.290 **		1.077 ***	1.185 *	1.220 **
				(0.139)		(0.523)		(0.142)	(0.560)	(0.431)
Nongreen Entrepreneurship					−7.257		−11.879	−2.053	−9.174	−10.407
					(5.204)		(7.991)	(1.179)	(5.704)	(6.070)
Interaction terms										
Green x										−0.739 *
Entrepreneurship Policy										(0.346)
Nongreen x										1.071 **
Entrepreneurship Policy										(0.483)
Controls										
Entrepreneurship			0.128			−0.043	0.012		−0.119	−0.796 **
Policy			(0.155)			(0.174)	(0.155)		(0.185)	(0.281)
Resources	−0.226		−0.134			−0.047	−0.068	0.008	−0.002	−0.011
	(0.195)		(0.207)			(0.167)	(0.193)	(0.131)	(0.173)	(0.186)
Population	0.120		0.022			0.227	0.259	0.146	0.394	0.434 **
	(0.113)		(0.278)			(0.177)	(0.263)	(0.085)	(0.254)	(0.191)
City Size	0.455		0.041			0.084	0.064	0.119	0.098	0.272
	(0.260)		(0.272)			(0.158)	(0.336)	(0.116)	(0.226)	(0.204)
Education	−0.137		−0.052			−0.342	−0.537	0.028	−0.693	−0.625
	(0.291)		(0.406)			(0.340)	(0.387)	(0.133)	(0.406)	(0.452)
Economic Activity	0.052		−0.046			0.025	−0.068	−0.013	0.003	−0.028
	(0.145)		(0.073)			(0.064)	(0.073)	(0.063)	(0.065)	(0.059)
Environmental Preservation	−0.070		−0.090			−0.091	−0.065	−0.050	−0.072	−0.024
	(0.094)		(0.135)			(0.095)	(0.122)	(0.062)	(0.089)	(0.111)
Basic Services	0.397		0.330			1.021	0.447	0.402	1.055	1.142
	(0.412)		(0.754)			(0.641)	(0.774)	(0.310)	(0.699)	(0.674)
Environmental			−0.045			−0.296	−0.314		−0.483 *	−0.253
Consciousness			(0.274)			(0.170)	(0.333)		(0.230)	(0.303)
Temporal Orientation			0.377			0.052	0.194		−0.063	−0.185
			(0.294)			(0.184)	(0.322)		(0.244)	(0.188)
Innovation Policy			−0.068			−0.154	−0.014		−0.105	−0.194 **
			(0.212)			(0.126)	(0.204)		(0.144)	(0.088)
Constant	0.018		−0.750	1.421 ***	−2.746	0.867	−4.126	0.078	−1.871	−2.348
	(0.923)		(0.894)	(0.177)	(1.969)	(1.078)	(2.569)	(0.687)	(2.230)	(1.952)
N	78		39	78	78	39	39	78	39	39
R^2^ within	0.061		0.145	0.773	0.128	0.387	0.247	0.807	0.447	0.625
R^2^ between	0.523		0.164	0.927	0.825	0.924	0.771	0.075	0.714	0.724
R^2^ overall	0.410		0.113	0.890	0.631	0.899	0.735	0.031	0.676	0.684

* *p* < 0.10, ** *p* < 0.05, *** *p* < 0.01.

## Appendix E

**Table A5:** Regression analysis (DV = environmental factors).

	1	2	3	4	5	6	7	8	9
Main independent variables									
Green Entrepreneurship			1.067 ***		1.182 ***		1.066 ***	1.115 ***	1.117 ***
			(0.151)		(0.320)		(0.162)	(0.322)	(0.328)
Nongreen Entrepreneurship				−6.368		−8.366	−0.526	−5.821	−5.848
				(5.344)		(6.707)	(2.687)	(6.028)	(5.996)
Interaction terms									
Green x									0.004
Entrepreneurship Policy									(0.229)
Nongreen x									−0.002
Entrepreneurship Policy									(0.320)
Controls									
Entrepreneurship		0.294 **			0.138	0.213 *		0.090	0.096
Policy		(0.113)			(0.107)	(0.117)		(0.117)	(0.224)
Resources	−0.185	0.013			0.093	0.060	0.036	0.121	0.121
	(0.275)	(0.172)			(0.156)	(0.150)	(0.110)	(0.135)	(0.148)
Population	0.024	−0.216			−0.027	−0.049	0.015	0.079	0.078
	(0.070)	(0.204)			(0.134)	(0.203)	(0.073)	(0.191)	(0.196)
City Size	0.298	0.307			0.346 **	0.323	−0.036	0.355 **	0.356 *
	(0.282)	(0.227)			(0.158)	(0.232)	(0.118)	(0.147)	(0.168)
Education	0.156	0.845 **			0.579 **	0.503	0.319 **	0.356	0.353
	(0.252)	(0.288)			(0.226)	(0.349)	(0.127)	(0.315)	(0.326)
Economic Activity	0.083	−0.023			0.043	−0.038	0.029	0.029	0.029
	(0.096)	(0.044)			(0.034)	(0.051)	(0.032)	(0.032)	(0.034)
Environmental Preservation	0.010	−0.015			−0.017	0.003	0.033	−0.004	−0.004
	(0.106)	(0.096)			(0.098)	(0.081)	(0.081)	(0.091)	(0.099)
Basic Services	−0.394	−0.884			−0.251	−0.802	−0.321	−0.229	−0.225
	(0.269)	(0.560)			(0.543)	(0.569)	(0.223)	(0.540)	(0.575)
Environmental		0.619 **			0.389	0.429		0.270	0.269
Consciousness		(0.272)			(0.252)	(0.248)		(0.321)	(0.349)
Temporal Orientation		0.366 *			0.068	0.237		−0.005	−0.003
		(0.171)			(0.207)	(0.158)		(0.167)	(0.177)
Innovation Policy		0.014			−0.065	0.051		−0.034	−0.036
		(0.159)			(0.088)	(0.136)		(0.091)	(0.109)
_cons	0.762	−0.470	1.359 ***	−2.409	1.012	−2.847	1.256	−0.725	−0.737
	(0.649)	(0.842)	(0.192)	(2.022)	(0.824)	(2.054)	(0.993)	(1.546)	(1.536)
*N*	78	39	78	78	39	39	78	39	39
R^2^ within	0.050	0.500	0.801	0.112	0.708	0.552	0.823	0.732	0.732
R^2^ between	0.275	0.004	0.853	0.751	0.874	0.736	0.860	0.636	0.637
R^2^ overall	0.214	0.018	0.839	0.590	0.866	0.699	0.842	0.589	0.590

* *p* < 0.10, ** *p* < 0.05, *** *p* < 0.01.
